# Allergies, body mass, and hospitalization due to arbovirus infection: A prospective surveillance study in Machala, Ecuador

**DOI:** 10.1017/S0950268823001656

**Published:** 2023-10-12

**Authors:** Anita S. Hargrave, Rachel Sippy, Cinthya Cueva, Mark Polhemus, Efrain Beltran, Mark A. Abbott, Anna M. Stewart-Ibarra

**Affiliations:** 1 Department of Internal Medicine, University of California San Francisco, San Francisco, California, USA; 2 Department of Internal Medicine, San Francisco VA Health Care System, San Francisco, California, United States; 3 Institute for Global Health and Translational Science, SUNY Upstate Medical University, Syracuse, New York, USA; 4 Department of Geography, University of Florida, Gainesville, Florida, United States; 5 Current affiliation: Department of Psychiatry, University of Cambridge, Cambridge, United Kingdom; 6 Faculdad de Medicina, Universidad Técnica de Machala, El Oro, Ecuador; 7 Department of Medicine, SUNY Upstate Medical University, Syracuse, New York, United States; 8 Inter-American Institute for Global Change Research, Montevideo, Uruguay

**Keywords:** arbovirus, body mass, allergies, hospitalization, dengue

## Abstract

Dengue, chikungunya, and Zika are arboviruses that cause 390 million infections annually. Risk factors for hospitalization are poorly understood. Communities affected by these diseases have an escalating prevalence of allergies and obesity, which are linked to immune dysfunction. We assessed the association of allergies or body mass with hospitalization for an arbovirus infection. From 2014 to 2017, we recruited participants with a clinical diagnosis of arbovirus infection. Arbovirus infections were laboratory-confirmed and allergies were self-reported. Mid-upper arm circumference (MUAC), weight, and height were measured. We used two logistic regression models to assess the relationships between hospitalization and allergies and between hospitalization and body mass (MUAC for participants <20 years old and body mass index (BMI) for adults ≥20 years old). Models were stratified by age group and adjusted for confounders. For allergies, 41 of 265 were hospitalized. There was no association between allergies and hospitalization. For body mass, 34 of 251 were hospitalized. There was a 43% decrease in hospitalization odds for each additional centimetre MUAC among children (aOR 0.566, 95% CI 0.252–1.019) and a 12% decrease in hospitalization odds for each additional BMI unit among adults (aOR 0.877, 95% CI 0.752–0.998). Our work encourages the exploration of the underlying mechanisms.

## Introduction

Latin America and Caribbean (LAC) countries are currently experiencing a rapid rise in chronic health conditions, such as obesity and allergies, as well as a growing epidemic of arboviral illness due to infections of dengue virus (DENV), chikungunya virus (CHIKV), and Zika virus (ZIKV). The dual burden of prevalently neglected tropical diseases paired with growing chronic conditions presents complex challenges to the public health systems and the people of LAC countries. There is relatively little information on how underlying chronic conditions affect arbovirus illness, which would provide public health guidance on prognoses among symptomatic patients presenting for clinical care.

DENV, CHIKV, and ZIKV are three common mosquito-borne pathogens that cause a substantial burden of disease globally. In LAC countries, infection, mortality, and morbidity from these arboviral diseases have been increasing [1–6]. In areas where these viruses are co-endemic, they are commonly diagnosed clinically without a confirmatory laboratory test and can be challenging to distinguish from one another as these infections often have a similar clinical presentation [7]. The viruses can cause a range of illnesses, from a short flu-like illness to chronic debilitating joint pain and life-threatening organ dysfunction [2, 5, 8]. Arbovirus co-infections also occur and are typically undetected without laboratory diagnostics. Hospitalization from arbovirus infection is often based on the clinical presentation and patient needs, without the confirmation of the aetiologic pathogen. It is estimated that approximately 500,000 people per year will require hospitalization from DENV alone and 2.5% of them will die [9]. Given the range of arboviral disease burden and overlapping clinical presentations, it is important to identify factors associated with hospitalization among patients with a symptomatic arbovirus infection who are presenting for clinical care.

LAC countries are also exhibiting an increased prevalence of allergies and obesity [10, 11]. Allergies are caused by an immune response to common environmental exposures. An inappropriate T-helper 2-cell response (T_H_2) leads to increased IgE mast cell activation and antibody production as well as the release of multiple inflammatory cytokines (e.g., IL-4, IL-6, IL-10, and IL-13) [12, 13]. In LAC countries, allergies are underdiagnosed and individuals are undertreated, meaning that they have repetitive or persistent exposure to their triggers [10, 14]. This may lead to chronic up-regulation of the T_H_2 response and a low-grade inflammatory state which could give way to more severe arbovirus symptoms [15]. Akin to allergic reactions, arboviral infections are associated with increased systemic inflammation and upregulation of T_H_2 cytokines [13, 16, 17]. During acute DENV infection, mast cells are strongly activated and may cause a dysfunctional immune response that can paradoxically lead to severe organ involvement and a higher viral load [17]. Despite this potential pathophysiology, it remains unclear whether allergies are associated with increased hospitalization from arbovirus infection.

Obesity rates are climbing in LAC countries. Among LAC countries, 37.8–60.0% of men and 50.4–66.7% of women were overweight or obese in 2013 [18]. Like allergies, obesity has been associated with persistent activation of pro-inflammatory cytokines and impaired immune function [19, 20]. The pathogenesis is under investigation; however, there are several described pathways. Obesity is related to an increased production of leptin, a hormone produced by fat cells. Leptin has been shown to cause increased production of pro-inflammatory proteins, macrophages, and natural killer lymphocytes. Concurrently, there is a decrease in adiponectin as body weight increases, which is an anti-inflammatory hormone that has the opposite effect as leptin [21, 22]. Other factors that may act independently or synergistically to drive an abnormal immune response include fatty-acid-induced inflammation, oxidative stress, endoplasmic reticulum stress, and adipose tissue hypoxia [20, 23]. Prior literature has suggested that obesity and allergies are associated with an increased risk of severe complications of arboviral infection [24–27]. Despite the high risk of these concurrent conditions in LAC countries, there are relatively few studies conducted in this region of the world, particularly in South America [28].

The aim of this study is to elucidate whether allergies or body mass exhibit an association with hospitalization from a symptomatic arbovirus infection in an endemic region in southern coastal Ecuador. Using data from a prospective surveillance study, we conducted separate analyses to assess the relationship between allergies, or increasing body mass, and hospitalization for acute arbovirus infection. Given the immune dysfunction associated with allergies and obesity, we anticipated that patients with allergies or those with higher body mass would be at increased risk of hospitalization.

## Methods

This study used data from a prospective arbovirus surveillance study. Detailed methods have been presented previously by Stewart-Ibarra *et al.* [4]. Between 2014 and 2017, 592 index cases with suspected arbovirus infections were recruited from clinical sites in Machala, Ecuador. Machala is a tropical coastal city in the southern region of Ecuador with a population of 280,694 inhabitants. It is the capital of El Oro Province and is an important agricultural hub within the country, particularly in the banana trade. The clinical sites that participated in our study were dispersed throughout the city of Machala; all participating sites were clinics/hospitals in the Ministry of Health (MoH) system, meaning that they can be utilized by anyone but are mainly used by persons without health coverage through the Ecuadorian Institute of Social Security (i.e., through employment) or those unable to pay out-of-pocket for private clinical services. Primary participating clinics included the main MoH hospital, Teófilo Dávila Hospital, and four MoH clinics: Brisas del Mar, Rayito de Luz, Mabel Estupiñan, and El Paraiso. Patients may have also entered the study via another MoH clinic in the area if they were subsequently referred to Teófilo Dávila Hospital. The data were collected by the same research staff member and the patient sociodemographic characteristics were similar across the clinical sites. Following a clinical diagnosis of an acute arbovirus infection by a MoH physician, symptomatic patients 6 months and older were invited to participate in the study. All participants provided consent and/or assent prior to enrolment. A study technician interviewed patients via survey, which included questions on health history, socioeconomic status, and home environment. A blood sample was collected, and specimens were processed at the State University of New York (SUNY) Upstate Laboratory in Machala where they were tested for DENV infection by the NS1 rapid test. Specimens collected in 2014 and 2015 were also tested by NS1 ELISA and IgM ELISA. Specimen aliquots were shipped to SUNY Upstate and were further tested using RT-PCR for DENV, CHIKV, and ZIKV infections. Patients were deemed arbovirus positive if one or more diagnostic tests were positive. Specific laboratory-confirmed diagnoses are available in Supplementary Table S1.

### Measurements

#### Allergies

Patients self-reported a history of chronic or current allergies and/or use of the allergy medication, loratadine, on the baseline questionnaire.

#### Body mass index and mid-upper arm circumference

Anthropometric measurements (weight, height, and mid-upper arm circumference (MUAC)) were measured at enrolment in the study. For participants under 20 years old, MUAC was used as a measure for nutritional status, as this is the recommended method for anthropometric assessment of body mass and nutritional status for this age group [29–35]. We used body mass index (BMI) for participants 20 years or older as BMI can be more rigorously interpreted using standard categories across genders, body types, and ages (i.e., <18.5 Underweight, 18.5–24.9 Healthy Weight, 25.0–29.9 Overweight, and ≥30.0 Obese) [36]. Weight and height were used to calculate BMI, dividing weight (kg) by height (meters squared) [36].

#### Hospitalization

Patients were deemed to be hospitalized if they had been admitted to the Teófilo Dávila Hospital for the treatment of their arbovirus infection. Teófilo Dávila Hospital is the principal tertiary public MoH hospital for the province of El Oro. It can receive patients from the entire province, but enrolled patients were from the city of Machala.

#### Demographic and clinical characteristics

Participants self-reported their age, gender, clinical symptoms, and medical history through the baseline questionnaire. Clinical symptoms included rash and two measurements of fever: self-reported fever within the last seven days and an oral temperature taken at study enrolment. Medical history was self-reported and included pregnancy and a history of diabetes.

### Statistical analysis

Separate analyses were conducted to assess the impact of increasing body mass or allergies on hospitalization among participants with a symptomatic arbovirus infection. Participant hospitalization was our primary outcome, defined as a binary variable (referent: not hospitalized). Allergy analyses were stratified into children and adolescents (0–19 years old) and adults (20 years and older). For the body mass models, participants were stratified into children (0–9 years old), adolescents (10–19 years old), and adults (20 years and older) to account for the differing standards of body mass measurements according to age. MUAC was used for children and adolescents while BMI was used for adults. Logistic regression models (generalized linear models) were used to assess the relationship between hospitalization and MUAC (for children and adolescents) or BMI (for adults) as well as hospitalization and self-reported allergies (for all age classes). BMI and MUAC were modeled as continuous variables. We adjusted for age and gender and reported both unadjusted odds ratios (uOR) and adjusted odds ratios (aOR). Confidence intervals (95% CI) were calculated using the profiled log-likelihood function. Data preparation and statistical analyses were conducted using R version 4.0.2 (R Foundation for Statistical Computing, Vienna, Austria) in RStudio version 1.1.423 (RStudio, Inc., Boston, MA).

## Results

### Demographics and hospitalization

**Figure 1.**
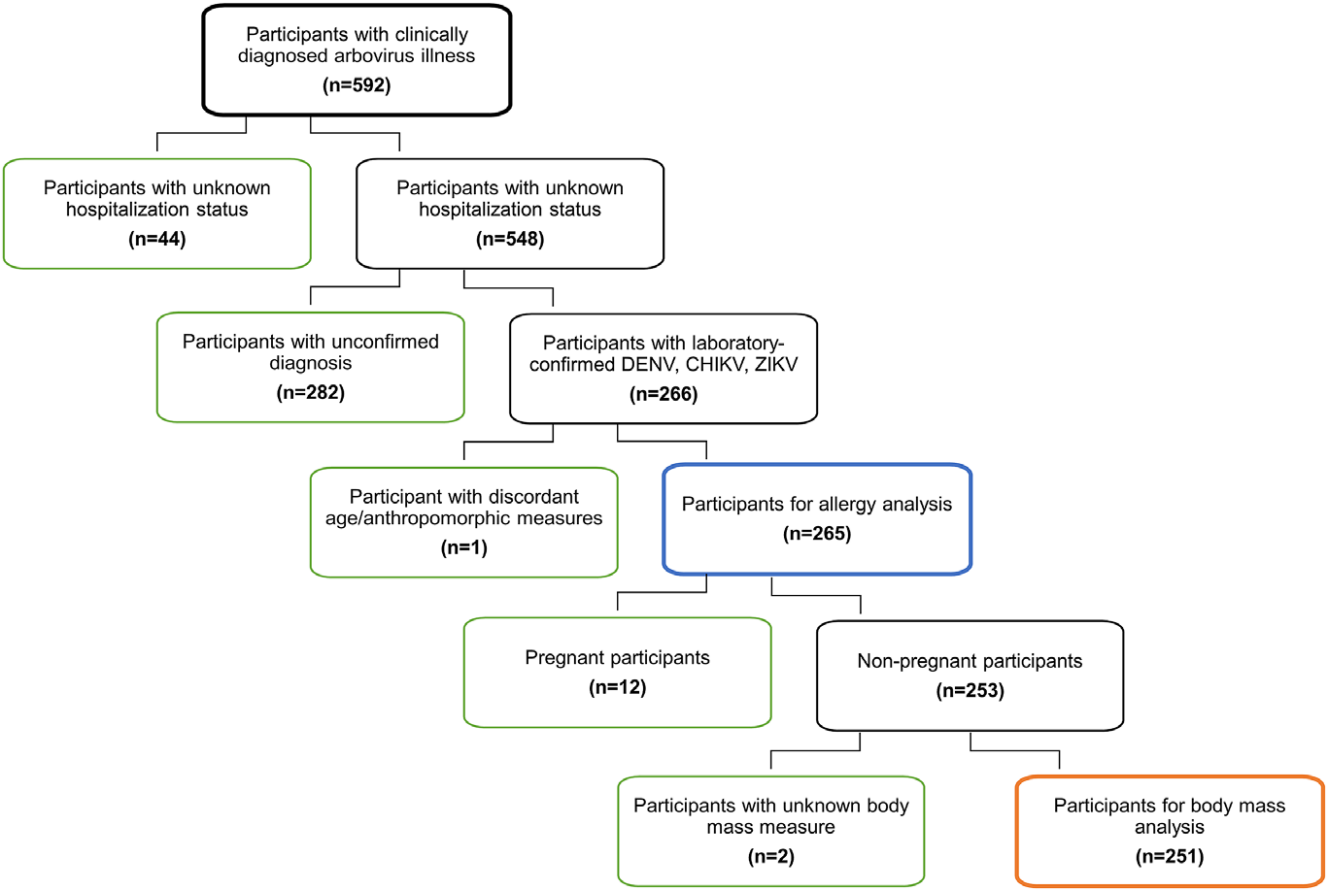
Flow chart of Selection for Allergy and Body Mass Analyses. Initial enrolment (bold black outline) included 592 participants; after exclusions, there were 265 participants for the allergy analysis (bold blue outline). Further exclusions left 251 participants for the body mass analysis (bold orange outline). DENV=dengue virus, CHIKV=chikungunya virus, ZIKV=Zika virus.

During the study period, we recruited 592 participants after referral by a MoH physician for symptomatology of an acute arbovirus infection. Of those referred, 266 participants had a confirmed diagnosis of acute DENV, CHIKV, or ZIKV infection by serum testing. We excluded one participant who had discordant age/body measurements and excluded 44 participants for unknown hospitalization status. This left 265 participants in our analysis of allergies. For analyses of body mass, we also excluded women who were pregnant (12 participants) given its impact on body mass and two participants with unknown body mass measures, leaving 251 participants (Figure 1).

In the allergy group, there were 142 participants aged 0–19 years old and 143 participants 20 years or older. In the body mass analyses, there were 39 participants aged 0–9 years old, 82 participants aged 10–19 years old, and 130 participants aged 20 years or older.

Demographics and clinical characteristics of study participants by hospitalization are shown in Table 1. Forty-one participants in the allergy group (15%) were hospitalized. Hospitalized participants were slightly younger than non-hospitalized participants and a lower proportion reported a rash (Table 1). More female participants (61%) were hospitalized than male participants (39%). The majority (80.5%) of hospitalized patients were enrolled at Hospital Teófilo Dávila. There were no differences in hospitalization according to oral temperature at the time of enrolment or fever in the last week. There were only six participants who reported having a history of diabetes. In the body mass group, 34 (13.5%) participants were hospitalized. The mean MUAC among participants aged 0–19 was 22.5 (range: 14.5–34 cm); none had an MUAC <12.5 cm, a common threshold for malnutrition [37, 38]. The mean BMI among participants 20 and older was 27.6 (range: 17.7–69.8); 61% had a BMI of 25 or greater, indicating that they were overweight or obese.

**Table 1.** Characteristics of study participants for allergy analysis and body mass analysis

Allergy Analysis		Hospitalized	Not Hospitalized
n		41	224
Primary exposure	History of allergies	9	34
		(22.0%)	(15.2%)
	Use of loratadine	0	9
		(0.0%)	(4.0%)
Subject demographics	Age, years	23.1	26.1
		(13.0)	(17.4)
	Male Gender	16	105
		(39.0%)	(46.9%)
Clinical symptoms	Temperature at enrolment,^a^ °C	37.1	37.4
		(0.8)	(0.8)
	Fever in the past seven days^b^	39	215
		(95.1%)	(95.6%)
	Rash^c^	12	71
		(29.3%)	(31.7%)
Medical history	Pregnant	7	5
		(17.1%)	(2.2%)
	Reported diabetes^d^	0	6
		(0.0%)	(2.7%)
Clinic^a^	Brisas del Mar	3	5
		(7.3%)	(2.2%)
	El Cambio	0	1
		(0.0%)	(0.4%)
	El Bosque	0	1
		(0.0%)	(0.4%)
	El Paraíso	0	58
		(0.0%)	(25.9%)
	Hospital Teófilo Dávila	33	52
		(80.5%)	(23.3%)
	Mabel Estupiñon	1	16
		(2.4%)	(7.1%)
	Puerto Bolivar	1	1
		(2.4%)	(0.4%)
	Patronato Municipal	1	0
		(2.4%)	(0.0%)
	Rayito de Luz	1	58
		(2.4%)	(25.9%)
	San Martin	1	0
		(2.4%)	(0.0%)
	Velasco Ibarra	0	31
		(0.0%)	(13.8%)
Body mass analysis		Hospitalized	Not hospitalized
N		34	217
Primary exposure	MUAC (children and adolescents only), cm	22.2	22.6
		(4.3)	(4.4)
	BMI (adults only)	24.5	27.9
		(3.3)	(7.3)
BMI classification (adults only)	Underweight (BMI <18.5)	0	2
		(0.0%)	(0.9%)
	Healthy Weight (18.5 ≤ BMI < 25)	9	39
		(26.5%)	(18.0%)
	Overweight (25 ≤ BMI < 30)	5	40
		(14.7%)	(18.4%)
	Obese (BMI ≥ 30)	1	34
		(2.9%)	(15.7%)
Subject demographics	Age, years	21.7	26.1
		(13.6)	(17.7)
	Male gender	16	104
		(47.1%)	(47.9%)

*Note:* Continuous variables are represented by mean and (standard deviation) while categorical variables are represented by n and (percentage of hospitalization group). Comprehensive characteristics are described for the allergy analysis, while characteristics for the body mass analysis are limited to covariates used in the analysis (i.e., age and gender), as the participants used for the body mass analysis are a subset of those used in the allergy analysis. In addition, BMI classifications are provided for informational purposes.

Abbreviations: BMI, body mass index; SD, standard deviation; MUAC, mid-upper arm circumference.

a Two missing observations.

b One missing observation.

c Two unknown responses.

d One missing observation and two unknown responses.

### Allergies and arboviral illness hospitalization

Results for the association between self-reported allergies and hospitalization for children, adolescents, and adults are in Table 2. After adjusting for age and gender, the odds of hospitalization among participants 0–19 years old with self-reported allergies was 1.717 (95% CI 0.541–5.037), and the odds of hospitalization among participants 20 years or older was 0.759 (95% CI 0.162–2.641). We did not find evidence of an association between allergies and hospitalization for participants in either age group.

**Table 2.** Allergies and hospitalization among children, adolescents, and adults

Age group	Factor	uOR	95% CI	aOR	95% CI
Children and Adolescents 0–19 years (n=122)	Self-Reported Allergies	1.605	0.516–4.562	1.717	0.541–5.037
Adults 20+ years (n=143)		0.966	0.211–3.239	0.759	0.162–2.641

*Note:* Results from unadjusted and adjusted models of the relationship between hospitalization and allergies for individuals within each age class. Adjusted models included age and gender covariates.

Abbreviations: uOR, unadjusted odds ratio; aOR, adjusted odds ratio; CI. confidence interval.

### MUAC, BMI, and arboviral illness hospitalization

Results for the association of body mass with hospitalization for children and adolescents are in Table 3. There was a 43% decrease in the odds of hospitalization for each additional centimetre of MUAC among children after adjusting for age and gender (aOR 0.566, 95% CI 0.252–1.019) (Table 2). However, the confidence intervals were too wide to rule out the possibility of no association. Among adolescents, there was no evidence of an impact of MUAC on hospitalization (aOR 0.974, 95% CI 0.785–1.189). Results for the association between body mass and hospitalization for adults are in Table 4. After adjusting for age and gender, we found a 12% decrease in the odds of hospitalization for each BMI unit (aOR 0.877, 95% CI 0.752–0.998).

**Table 3.** Mid-upper arm circumference and hospitalization among children or adolescents

Age group	Factor	uOR	95% CI	aOR	95% CI
Children 0–9 years (n=39)	MUAC (cm)	0.905	0.628–1.208	0.566	0.252–1.019
Adolescents 10–19 years (n=82)		0.984	0.827–1.163	0.974	0.785–1.189

*Note:* Results of unadjusted and adjusted models of the relationship between hospitalization and MUAC among children (0**–**9 years) or adolescents (10**–**19 years). Adjusted models included age and gender covariates.

Abbreviations: uOR, unadjusted odds ratio; aOR, adjusted odds ratio; CI, confidence interval; MUAC, mid-upper arm circumference.

**Table 4.** Body mass index and hospitalization among adults

Factor	uOR	95%CI	aOR	95%CI
BMI	0.887	0.778–0.991	0.877	0.752–0.998

*Note:* Results from unadjusted and adjusted models of the relationship between hospitalization and BMI for adults (n=130). Adjusted models included age and gender covariates.

Abbreviations: uOR, unadjusted odds ratio; aOR, adjusted odds ratio; CI, confidence interval, BMI, body mass index.

## Discussion

In this study, we analyzed the association between hospitalization from symptomatic arbovirus infection and allergies or body mass among participants with confirmed acute DENV, CHIKV, and ZIKV infection. We conducted our study in coastal Ecuador, which is experiencing a simultaneous increase in obesity, allergies, and arbovirus infection, yet remains an understudied region [4, 28, 39]. The analyses for each health condition were motivated by their association with variations in immune function that may impact the prognosis of arbovirus infection, precipitating a higher level of care. Our study did not find that allergies were associated with hospitalization risk and analyses showed modest decreases in hospitalization risk for children and adults with increasing measures of body mass. Although the confidence intervals are too wide to exclude a null association among children or adolescents, results showed possible evidence of decreased hospitalization among adults with increasing body mass as measured by BMI. The direction of this association was unexpected; the pathophysiology of arbovirus infection is complex and this highlights the need for ongoing research to better define how chronic inflammatory states impact the need for hospitalization.

### Allergies and hospitalization

Allergies have been associated with complications from arboviral infection, particularly from DENV [25, 28, 40]. Our results showed no association between allergies and hospitalization among participants with symptomatic arbovirus infection in any age group. Chronic conditions are often underdiagnosed in regions of Ecuador [41]; it is possible that the true prevalence of allergies in our population is not accurately reflected by a self-reported history of allergies or use of allergy medication. This possibility is supported by the small number of participants reporting a history of diabetes. Given the number of overweight or obese participants within our study cohort, it is unlikely that so few would have diabetes. Many participants may not know or have been diagnosed with diabetes. Although there are many other methods of measuring these conditions, prior literature has used self-reported medical history as a robust measure of allergies and diabetes [25]. Future research should utilize a more thorough evaluation of allergy status among participants.

### MUAC and hospitalization in children

Previous studies of body mass among children and severe arbovirus infection have yielded mixed results: a meta-analysis of studies using clinical manifestations of DENV infection (dengue fever, dengue haemorrhagic fever, or dengue shock syndrome) as measures of severity found no change in dengue severity among malnourished or overweight/obese children [42]. A more recent meta-analysis, again using clinical manifestations of DENV infection as measures of severity, found a 38% increase in odds of severe dengue infection among obese participants compared to non-obese participants [43]. Our findings did not show an association between body mass and hospitalization for arbovirus infection; the wide uncertainty for the effect estimate suggests a lack of power. None of the participants in our study developed dengue shock syndrome or dengue haemorrhagic fever; the association between obesity and severe DENV infection may only exist for the most extreme clinical presentations of DENV infection. Infections have been shown to lead to anorexia, altered metabolic consumption, and poor dietary absorption among children [44], meaning that those with a reserve of adipose tissue could benefit from some infectious processes.

Increased MUAC may be a surrogate measure for improved micronutrient intake. Many micronutrients influence immunomodulation through oxidative stress [45, 46]. In Ecuador, serum ferritin and retinol-binding protein concentrations have been associated with apparent DENV infection after adjusting for an acute-phase reaction [45, 47]. Zinc is another trace element that plays a key role in DNA replication, RNA transcription, and cellular replication, vital to the development of innate immunity. Zinc deficiency is common in low-income countries and has been associated with a more severe arboviral infection [48]. More comprehensive evaluations will be needed to understand whether immune function or nutritional status mediates the relationship between obesity and severity of arboviral infection and if this applies across the full spectrum of clinical manifestations of arboviral infection.

### MUAC and hospitalization in adolescents

Our results did not show evidence of an association between MUAC and hospitalization among adolescents with a symptomatic arbovirus infection. There is a dearth of literature on hospitalization of adolescents with arbovirus infection; therefore, the impact of body mass on hospitalization remains unclear [43]. However, adolescence and early adulthood have been identified as high-risk periods for developing a clinically significant dengue infection [49, 50]. Therefore, it is important to continue identifying potential risk factors for severe diseases in this relatively understudied age group.

### BMI and hospitalization in adults

Previous studies have shown increased BMI to be associated with the severity of arbovirus infection, including the duration of hospitalization [26] and chronic arthritis [51]. Our results indicate that increasing BMI had a protective association against hospitalization for arboviral infection. Although our study has limitations, the uncertainty is precise; the results suggest that increased BMI may be associated with decreased odds of hospitalization. Obesity paradox is a phenomenon that has been observed in diseases that induce cachexia and among patients with multiple chronic diseases [52, 53]. It is thought that adipose reserves are beneficial in catabolic states and may have immune benefits in selective populations, particularly older adults [53, 54]. Central obesity also is more strongly associated with low-grade inflammatory states and higher morbidity and mortality than peripheral obesity [53]. Further, overweight patients with increased cardiorespiratory fitness have been found to have *better* life expectancy [53]. Finally, studies have shown that Ecuador is not only impacted by excess body weight but also by decreased height and stunted growth [39, 55]. Therefore, elevated BMI measurements in our study may have been driven by the *combination* of increased body weight and shortened height for many of our participants. Ultimately, this data suggests that the health implications of increasing BMI may be more contingent on characteristics such as age, distribution of fat tissue, activity level, and cachectic disease state.

### Limitations

We acknowledge several limitations in our study. Mid-upper arm circumference is primarily validated for use in ages 6 months to 5 years of age to detect severe malnutrition. There is a debate regarding its utility outside of this age range. Therefore, MUAC may not be the best surrogate marker of body mass, particularly if it is not correlated with sex and year of age [29]. BMI is similarly an imperfect measure of body mass, given that it does not detect differences between adipose tissue, muscle, or bone, nor does it evaluate the distribution of fat (e.g., central versus peripheral adipose tissue) [56]. Although other methods of measurement exist, they are often expensive and require highly trained staff to ensure standardization across measurements and equipment [36]. BMI is the most commonly used measure of body fat, allowing us to compare our results to previous studies while using standardized categories of weight endorsed by the World Health Organization and the Center for Disease Control [36, 57]. BMI has also been shown to correlate with the risk of weight-related morbidity and with more direct measures of body fat such as underwater weight [56, 57]. Ultimately, MUAC and BMI offer low-cost, low-barrier methods for measuring the nutritional status or weight when more expensive and time-intensive measures cannot be obtained, but these are only proxy measures for the true immunological and nutritional status of participants. Understanding the relationship between obesity and clinical presentations of arboviral infection will require a more in-depth evaluation of immune markers and nutritional status.

We did not detect statistically significant differences in hospitalization among children or adolescents, which may be due to the lack of power to detect a modest association among children or suboptimal body mass measures for adolescents. We did not have a sufficient sample size to evaluate each type of arbovirus separately, and although arboviruses are often clinically diagnosed and triaged without laboratory confirmation of the subtype [7], there may be variation in the impact of obesity and/or allergies on hospitalization by arbovirus type. Finally, there are numerous potential confounders in the relationship between weight, allergies, and hospitalization, depending on the exact causal mechanisms at play. Measurements for many of these confounders were unavailable to us in this dataset. Future research including these confounders and among large diverse populations will be necessary to better elucidate the relationship between our exposures of interest and hospitalization.

## Conclusions

The world is experiencing an unprecedented rise of chronic inflammatory conditions in areas that are simultaneously experiencing epidemics of mosquito-transmitted viral infections. In this study of participants with a confirmed symptomatic arbovirus infection, we found that increasing body mass exhibited a protective association against hospitalization for adults. However, our confidence intervals were too wide to exclude a null association among children or adolescents. Self-reported allergies did not impact hospitalization. Our research suggests that further research of the underlying mechanisms is needed.

## Supporting information

Hargrave et al. supplementary material Hargrave et al. supplementary material

## Data Availability

All data and code have been deposited here: https://github.com/rsippy/Dengue-BMI.

## Supplementary material

For supplementary material accompanying this paper visit http://doi.org/10.1017/S0950268823001656. click here to view supplementary material
