# Supplementary material for: Allergies, body mass, and hospitalization due to arbovirus infection: A prospective surveillance study in Machala, Ecuador
Source: Epidemiol Infect. 2023 Oct 12;151:e181. doi: 10.1017/S0950268823001656 (PMC10644055; doi:10.1017/S0950268823001656)
Supplement: Hargrave et al. supplementary material [file S0950268823001656sup001.docx]

| **Supplementary Table S1: Arbovirus Laboratory Confirmation** | | |
| --- | --- | --- |
| **Diagnosis** | **Hospitalized** | **Not Hospitalized** |
| Dengue only | 30  (73.2%) | 110  (41.9%) |
| Dengue and chikungunya | 0  (0.0%) | 1  (0.4%) |
| Chikungunya only | 9  (22.0%) | 100  (37.7%) |
| Zika only | 2  (4.9%) | 13  (4.9%) |
| Results are represented as n and (percentage of hospitalization group). Dengue was laboratory-confirmed if the sample was positive by NS1 rapid test, NS1 ELISA, IgM ELISA, or RT-PCR. Chikungunya was laboratory confirmed if the sample was positive by RT-PCR. Zika was laboratory confirmed if the sample was positive by RT-PCR. | | |
